# Pathogenicity characteristics of different subgenotype pseudorabies virus in newborn piglets

**DOI:** 10.3389/fvets.2024.1438354

**Published:** 2024-08-07

**Authors:** Lei Xu, Qian Tao, Tong Xu, Yanting Yang, Yang Zhang, Zheyan Liu, Yuancheng Zhou, Ling Zhu, Zhiwen Xu

**Affiliations:** ^1^Key Laboratory of Animal Diseases and Human Health of Sichuan Province, College of Veterinary Medicine, Sichuan Agricultural University, Chengdu, China; ^2^Livestock and Poultry Biological Products Key Laboratory of Sichuan Province, Sichuan Animal Science Academy, Chengdu, China; ^3^Animal Breeding and Genetics Key Laboratory of Sichuan Province, Sichuan Animal Science Academy, Chengdu, China

**Keywords:** pseudorabies, pathogenicity, variant strains, isolation, inflammatory factor

## Abstract

Pseudorabies virus is a major pathogen in the pig industry, causing substantial economic losses. The emergence of pseudorabies virus variant strains in China has led to extensive spread, raising concerns about their potential impact. However, the differences in pathogenicity between the classical strains and the variant strains of genotype II are not well understood. In this study, we isolated three pseudorabies virus strains to evaluate their replication characteristics and to examine the differences in virulence genes among various subgenotypes strains. Additionally, a piglet infection model was utilized to investigate the clinical features of infection, tissue tropism, and the inflammatory responses induced by these strains. Our results showed that the genotype II variant strains (MS, XJ, LS, and CZ) had significantly larger plaque sizes and higher replication capacities than the genotype II classical strain Fa. The animal experiments revealed significant differences in pathogenicity among the pseudorabies virus subgenotype strains, with the variant strains showing higher mortality rates, more severe clinical symptoms, increased nasal virus shedding, and a more robust inflammatory response compared to the genotype II classical strain. There were also notable differences in tissue tropism among the strains. In terms of tissue viral loads, the genotype II variant strains did not exhibit a significant advantage over the genotype I classical strain. Furthermore, our findings indicate that antibodies against the genotype II classical strains have a reduced neutralizing capacity against the genotype II variant strains. On the other hand, antibodies against the genotype II variant strains displayed similar neutralizing abilities against both classical and variant strains. Overall, these findings offer important insights into the distinctions among pseudorabies virus subgenotypes and their implications for the clinical control of pseudorabies virus infections in pig farming.

## 1 Introduction

Pseudorabies (PR), also known as Aujeszky’s disease (AD), is an acute infectious disease caused by the pseudorabies virus (PRV), which belongs to the order *Herpesvirales*, family *Herperviridae*, subfamily *alphaherpersvirinae*, and genus Var*icellovir*us (1).^1^ PRV is transmitted primarily through direct contact with infected suids or by consuming raw offal from infected pigs, whether wild or domestic, or pig carcasses (2). PRV strains are currently classified into two genotypes: genotype I and genotype II. Genotype I is commonly found in Argentina, Spain, Italy, the United States, and China (3–6), whereas genotype II PRV isolates are predominantly present in China. Genotype II is further categorized into two subgenotypes: the classic strains and variant strains (7). Infection with PRV leads to acute and fatal infections in piglets, abortion in sows and growth retardation or weight loss in growing pigs (8, 9). While PRV can infect various domestic and wild animals, pigs are its only natural host (10, 11). In recent years, several cases have confirmed that PRV can also cause encephalitis in humans, leading to symptoms such as fever, sweating, weakness, seizures, and even death (12). The recovery prospects for patients are extremely poor, posing a severe threat to public health (13).

Before 2011, China had effectively controlled PRV through vaccination using the Bartha-Hungary-1961 strain-based vaccine (Bartha-K61). Some regions, like parts of the United States, Canada, and Europe, even achieved complete eradication of PRV (14). However, from October 2011, a rapid spread of the PRV epidemic occurred in China, causing substantial economic losses to the pig industry. The outbreak was caused by the genotype II PRV variant strain, which differs from classical strains due to deletions in amino acid positions 75–77 of the glycoprotein B (gB) (15). It is worth noting that the Bartha-K61 vaccine has been found ineffective against the genotype II variant strain (16). Since its emergence, the PRV variant strain has caused significant economic losses to the pig industry in China (17).

Variant strains of PRV, in comparison to the classical PRV strains, display enhanced virulence, increased infectivity, faster transmission, and present with more severe clinical symptoms. Recent studies have even indicated increased virulence in recombinant strains (18). However, the relationship between PRV genetic features and virulence is still not fully understood. In this study, we isolated variant strains of LS, MS, and CZ. The early classical strain Fa and the variant strain XJ were used as reference strains to analyze the genetic evolution and virulence changes of the current popular variant strains, focusing on the main glycoprotein genes gB, glycoprotein C (gC), glycoprotein D (gD), and glycoprotein E (gE) and PRV pathogenicity to piglets.

## 2 Materials and methods

### 2.1 Virus and cells

Baby hamster kidney (BHK-21) cells were cultured in Dulbecco’s modified Eagle’s medium (DMEM, Gibco, Waltham, MA, United States), supplemented with 10% fetal bovine serum (FBS, Gibco, Waltham, MA, United States), and maintained in an incubator with 5% CO_2_. The PRV XJ strain was isolated and preserved in our laboratory, as previously described (19). The PRV Fa strain was also preserved in our laboratory (20). Pig anti-Fa hyperimmune serum and pig anti-XJ strain hyperimmune serum were prepared and stored in our laboratory.

### 2.2 Isolation and identification of PRV

The polymerase chain reaction (PCR) method was utilized for the detection of PRV in brain, liver, and lung tissues collected from pigs from farms located in different regions of Sichuan Province. The PCR was conducted using the following primers: gE forward primer (5′-ATCTGGACGTTCCTGCCC-3′) and gE reverse primer (5′-GTAGATGCAGGGCTCGTACA-3′) (21). The PRV-positive samples were minced and homogenized in 1 mL of DMEM. Subsequently, the PRV-positive samples underwent three freeze–thaw cycles and were centrifuged at 5000 rpm for 10 min at 4°C to obtain the supernatant. Utilize the PCR method to confirm the presence of PRV in the supernatant (21). The supernatant from PRV-positive samples was then filtered through a 0.22 μm membrane. The filtered supernatant was used to inoculate confluent BHK-21 cells monolayers. Following a 1-h incubation at 37°C, the supernatant was aspirated, and the cells were further cultured in DMEM with 2% FBS for 48 h. Cytopathic effects (CPE) were monitored daily. Infected cells were fixed using 4% paraformaldehyde and then characterized by immunofluorescence assay (IFA) with a monoclonal antibody against the PRV gB protein sourced from Beijing TianTech Biotechnology (19).

### 2.3 Transmission electron microscopic observation of virions

At 48 h post-infection (hpi), the cells were harvested and fixed using a 3% glutaraldehyde fixative. Following fixation, the cells were processed for transmission electron microscopy by Chengdu Lilai Biotechnology Co., Ltd., enabling the capture of virions images (21).

### 2.4 One-step growth curve

As described previously (22), one-step growth kinetics assays were conducted. BHK-21 cells were plated in 96-well plates at a density sufficient to form confluent monolayer. The cells were then infected with the PRV strains MS, CZ, XJ, LS, or Fa at a multiplicity of infection (MOI) of 0.1. Post-infection, cells and supernatants were harvested at intervals of 6, 12, 24, 36, 48, and 72 hpi. Virus titers were calculated using the 50% tissue culture infectious dose (TCID_50_) assay, and these data were utilized to generated the one-step growth curves for each PRV strain.

To evaluate plaque sizes of PRV strains from different subgenotype, BHK-21 cells were infected with PRV at a titer of 100 TCID_50_, employing plaque assays according to the previous study protocol (21).

### 2.5 Phylogenetic analysis

Total DNA from the XJ, MS, CZ, LS and Fa strain was extracted using a universal genomic DNA kit (Cofitt, Jiangsu, China). PCR amplification was conducted using PrimeSTAR HS DNA Polymerase and GC Buffer (TaKaRa Bio, Shiga, Japan), with primers for each gene listed in Table 1. Purified positive PCR products were cloned into the pUCm-T vector and submitted to Shanghai Bioengineering Co., Ltd. for sequencing. Full-length sequences of the gB, gC, gD, and gE genes from the MS, CZ, XJ, LS, and Fa strains were aligned using DNAStar version 15.7 software. Phylogenetic trees were constructed based on the major virulence genes (gB, gC, gD, and gE) using Molecular Evolutionary Genetics Analysis (MEGA; version 5.1) software, employing the maximum likelihood method and 1,000 bootstrap replicates for validation.

**Table 1:** Primers for gene sequences.

Primer name	Sequence	Size
gB-1F	ATGCCCGCTGGTGGCGGTCTT	1,433 bp
gB-1R	GCGTACAGCTGCGCCAGCTCG	
gB-2F	TTCGTGGTGGCCTTCCGCCCG	1,365 bp
gB-2R	CTAGGGGGCGTCGGGGTCCTC	
gC-F	ATGGCCTCGCTCGCGCGTGCG	1,464 bp
gC-R	TCACAGCGCGGACCGGCGGTA	
gD-F	ATGCTGCTCGCAGCGCTATTG	1,215 bp
gD-R	CTACGGACCGGGCTGCGCTTT	
gE-F	ATGCGGCCCTTTCTGCTGCGC	1,740 bp
gE-R	TTAAGCGGGGCGGGACATCAA	

### 2.6 Animal experiments

Thirty two-week-old female piglets were screened and confirmed to be negative for PRV gB-specific antibodies via an enzyme-linked immunosorbent assay (ELISA) kit (ID. vet, France) and negative for PRV DNA using PCR with gE-specific primers. They were then randomly assigned into 6 groups, each group consisting of 5 piglets. Piglets in groups 1 to 5 (*n* = 5 each) were intranasally infected with 2 mL of MS, CZ, Fa, XJ, or LS strains, at a titer of 10^7^ TCID_50_/mL, respectively. Group 6 served as the uninfected control and was intranasally inoculated with 2 mL of DMEM. Rectal temperatures and clinical signs were recorded daily following inoculation. Daily nasal swabs collections were conducted, and viral shedding was assessed using qRT-PCR. Clinical symptoms were scored according to previous descriptions as follows (23): (1) temperature elevation above 40°C; (2) temperature elevation above 40°C with respiratory distress; (3) ataxia; (4) convulsions; and (5) moribund or dead.

Following the challenge, piglets exhibiting distress symptoms, including respiratory difficulties, rapid or irregular breathing, inability to stand, or anorexia, were humanely euthanized using a lethal dose of pentobarbital sodium (40 mg/kg). On day 14, all remaining piglets, including the survivors and control group piglets, were euthanized via an intrajugular injection of pentobarbital sodium (40 mg/kg). After euthanasia, tissues including the cerebellum, brainstem, tonsils, inguinal lymph nodes, mesenteric lymph nodes, lungs, liver, brain, intestines, spleen, and kidneys were collected for further analysis.

### 2.7 Real-time fluorescence quantitative PCR (qRT-PCR)

Fresh tissues samples and nasal swabs were collected for viral loads analysis. Genomic DNA was extracted from the samples using a universal genomic DNA kit (Cofitt, Jiangsu, China). We performed qRT-PCR using TB Green^®^ Premix Ex Taq^™^ (Tli RNaseH Plus) (RR420Q, Takara, Japan) to quantify the PRV DNA copies, specifically for the gE gene with the following primers: Forward (5’-CTTCCACTCGCAGCTCTTCT-3′), Reverse (5’-TAGATGCAGGGCTCGTACAC-3′). The gene copies per gram of tissue sample were expressed as log_10_ copies.

### 2.8 Cross-neutralization assay

Neutralizing titers of antisera obtained from Fa vaccine and rPRV-gE/gI/TK^−^ strain-inoculated animals were determined using methods previously described (24). The sera were first heat-inactivated at 56°C for 30 min prior to serial dilution. Subsequently, the diluted sera were incubated with an equal volume of XJ, LS, Fa, CZ, or MS, each at a titer of 200 TCID_50_/0.1 mL, at 37°C for 1 h. Next, 100 μL of BHK-21 cells, prepared at a concentration of 10^6^ cells/mL in DMEM with 2% FBS, were added to each well of the 96-well plates. The plates were incubated at 37°C with 5% CO_2_, and CPE were monitored daily. Neutralizing antibody titers were calculated using the Reed-Muench method, defining the serum dilution preventing 50% of cells from CPE as the neutralization titer.

### 2.9 Quantification of inflammatory markers

Concentration of IL-1*β*, IL-6, IL-8, and TNF-*α* were measured in lung, brain, spleen, and intestine tissues using Elisa kits from Thermo Fisher, according to the manufacturer’s protocol.

### 2.10 Statistical analysis

All data were analyzed using GraphPad 7.04 software with one-way analysis of variance (ANOVA). All results are presented as the mean ± standard deviation. A significance level of *p* < 0.05 was considered statistically significant.

## 3 Results

### 3.1 *In vitro* proliferation characteristics of PRV

**Figure 1:**
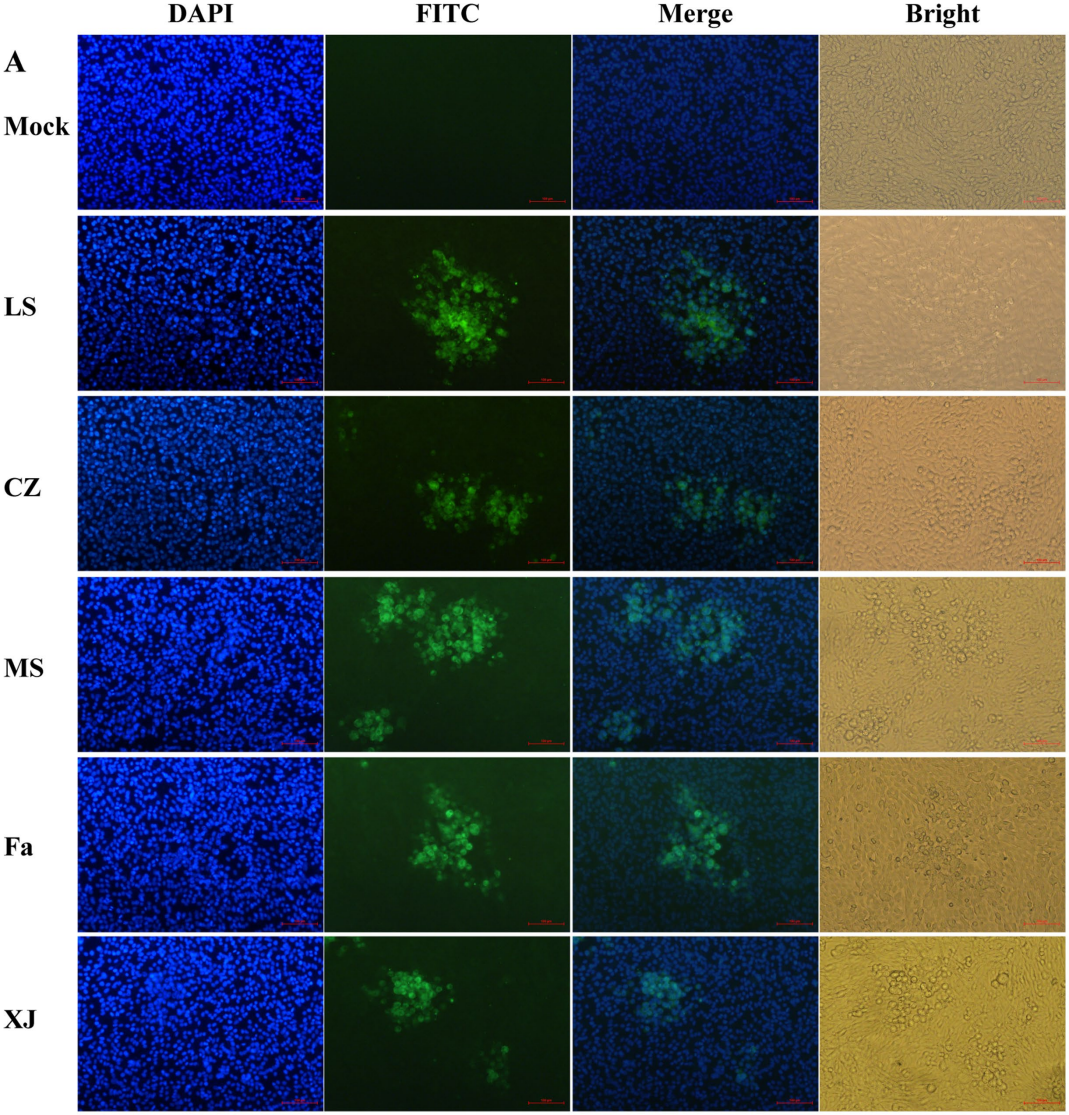
IFA to identify PRV strains. 4′,6-diamidino-2-phenylindole (DAPI): DAPI was utilized for staining cell nuclei. Fluorescein Isothiocyanate (FITC): following binding of the anti-gB protein mouse monoclonal antibody to the PRV gB protein, FITC-labeled goat anti-mouse immunoglobulin G (IgG) is utilized to bind to the complex, resulting in the emission of green fluorescence. Merge: combine DAPI staining pictures and FITC pictures. Bright: capture images of cells under bright field.

**Figure 2:**
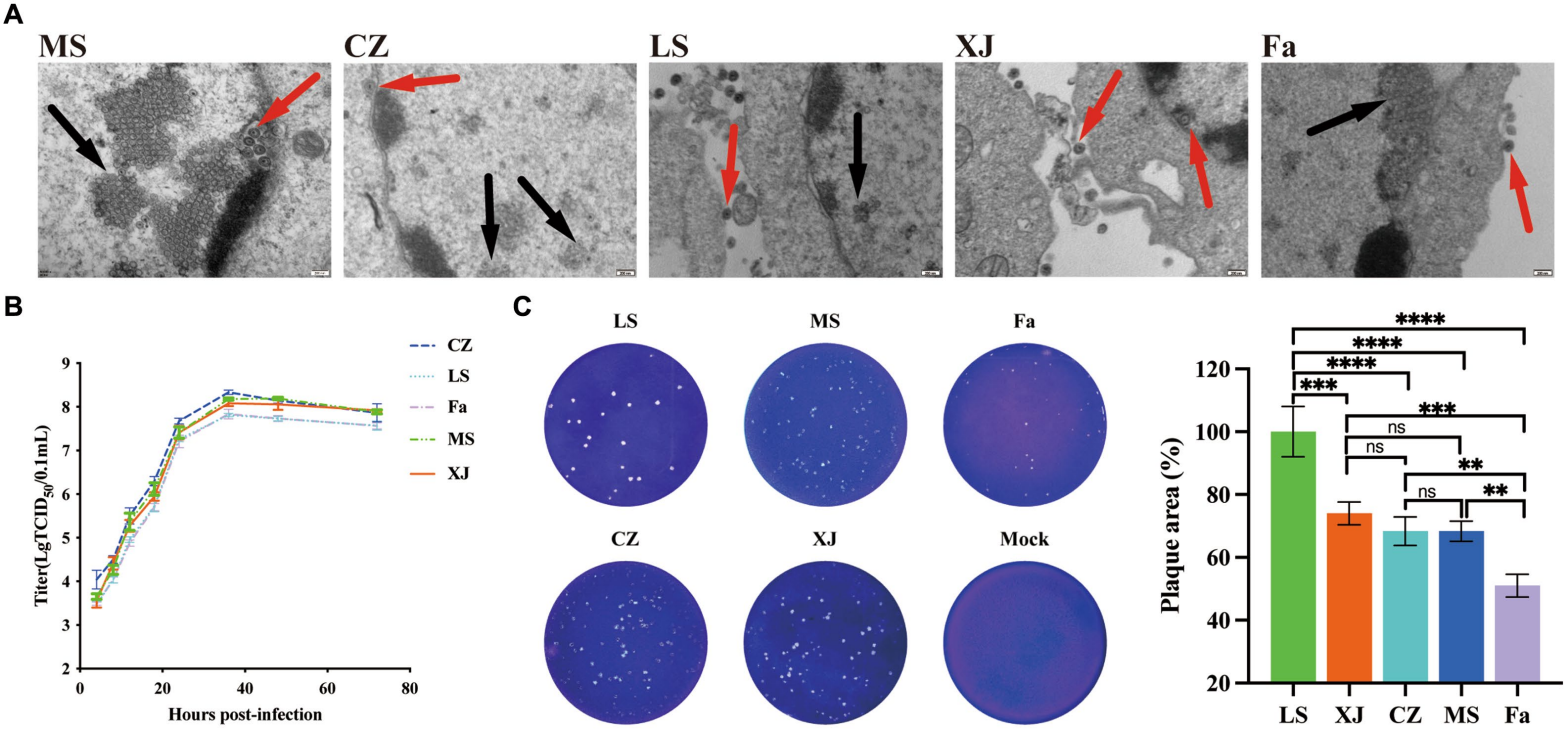
Biological characteristics of different strains of PRV *in vitro*. **(A)** Transmission electron microscopy images of PRV. The red arrow points to the mature virion, and the black arrow points to the noncapsular nucleocapsid. **(B)** One-step growth curves of the LS, XJ, MS, CZ, and Fa strains in BHK-21 cells. In the growth curves, the x-axis represents the infection time, and the y-axis represents the virus titer. **(C)** BHK-21 cells were infected with the LS, XJ, MS, CZ and Fa strains. The plaque size of each virus was assessed by performing the plaque assay. ** *p* < 0.01; *** *p* < 0.001; **** *p* < 0.0001; ns, not significant.

Following PRV infection, a distinct green fluorescence signal, indicative of the PRV gB protein, was detected within the CPE regions (Figure 1). Transmission electron microscopy showed virus particles, devoid of a capsule structure, within the nucleus, measuring approximately 100 nm in diameter (Figure 2A). Furthermore, mature virus particles, enclosed by a capsule, were identified in the cytoplasm, measuring about 160 nm in diameter. These observations are consistent with the known surface characteristics of PRV. The isolated strains, MS, CZ, and LS, were named according to their geographical origins. The one-step growth curve demonstrated that the MS, CZ, LS, XJ, and Fa strains exhibited similar growth characteristics in BHK-21 cells, peaking at 36 hpi with virus titers of 10^8.18^/mL for MS, 10^8.33^/mL for CZ, 10^8.08^/mL for XJ, 10^7.81^/mL for LS, and 10^7.83^/mL for Fa, as illustrated in Figure 2B. Plaque assay results showed that the LS strain had a significantly higher plaque generation ability compared to the MS, XJ, CZ, and Fa strains (Figure 2C). The MS, XJ, and CZ strains exhibited similar plaque generation abilities, each significantly exceeding that of the Fa strain (Figure 2C). This indicates that the infectivity of the LS, MS, XJ, and CZ strains was significantly stronger than that of the Fa strain.

### 3.2 Phylogenetic analysis

The gB, gC, gD, and gE gene sequences of the MS, CZ, and LS strains were uploaded to NCBI (accession numbers: OR338844-OR338846 and OR334583-OR334586).

**Figure 3:**
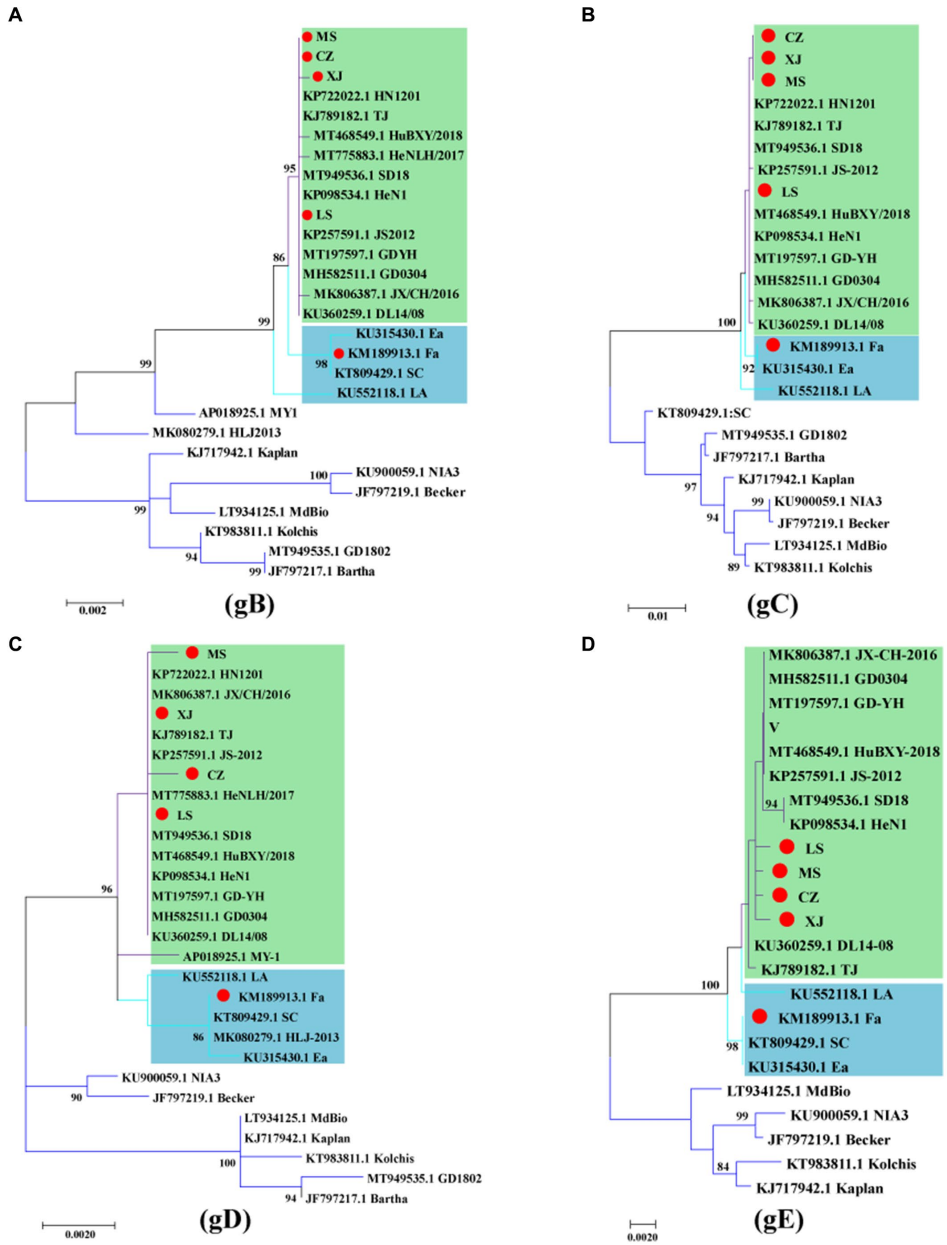
Phylogenetic analysis of the gB, gC, gD and gE genes of PRV. Phylogenetic trees based on the gB **(A)**, gC **(B)**, gD **(C)** and gE **(D)** genes were constructed using the maximum likelihood method. The PRV strains used in this study are indicated by a red dot. The PRV genotype II classical strains are indicated by green, and the PRV genotype II variant strains are indicated by blue.

Analysis of the gB and gC gene sequences indicated that the MS, CZ, and LS strains are significantly distinct from the genotype II classical strains in China and genotype I strains from other countries, clustering more closely with the variant strain XJ. Within this cluster, the MS and CZ strains showed a close relationship with HN1201, whereas the LS strain showed greater similarity to JS2012, all of which are distinct from the classical strains Fa and Ea, as illustrated in Figures 3A,B. Analysis of the gD sequences revealed that the MS strain shared similarity with HN1201, the CZ strain had a close relationship with HeNLH/2017, and the LS strain was closely associated with SD2018 (Figure 3C). Subsequentanalysis of gE gene sequences indicated that the MS, CZ, and LS formed a distinct cluster within the genotype II variant strains (Figure 3D). Altogether, the phylogenetic analysis of the gB, gC, gD, and gE genes suggests that the MS, CZ, and LS strains are classified as genotype II variant strains.

### 3.3 Clinical symptoms and viral excretion

**Figure 4:**
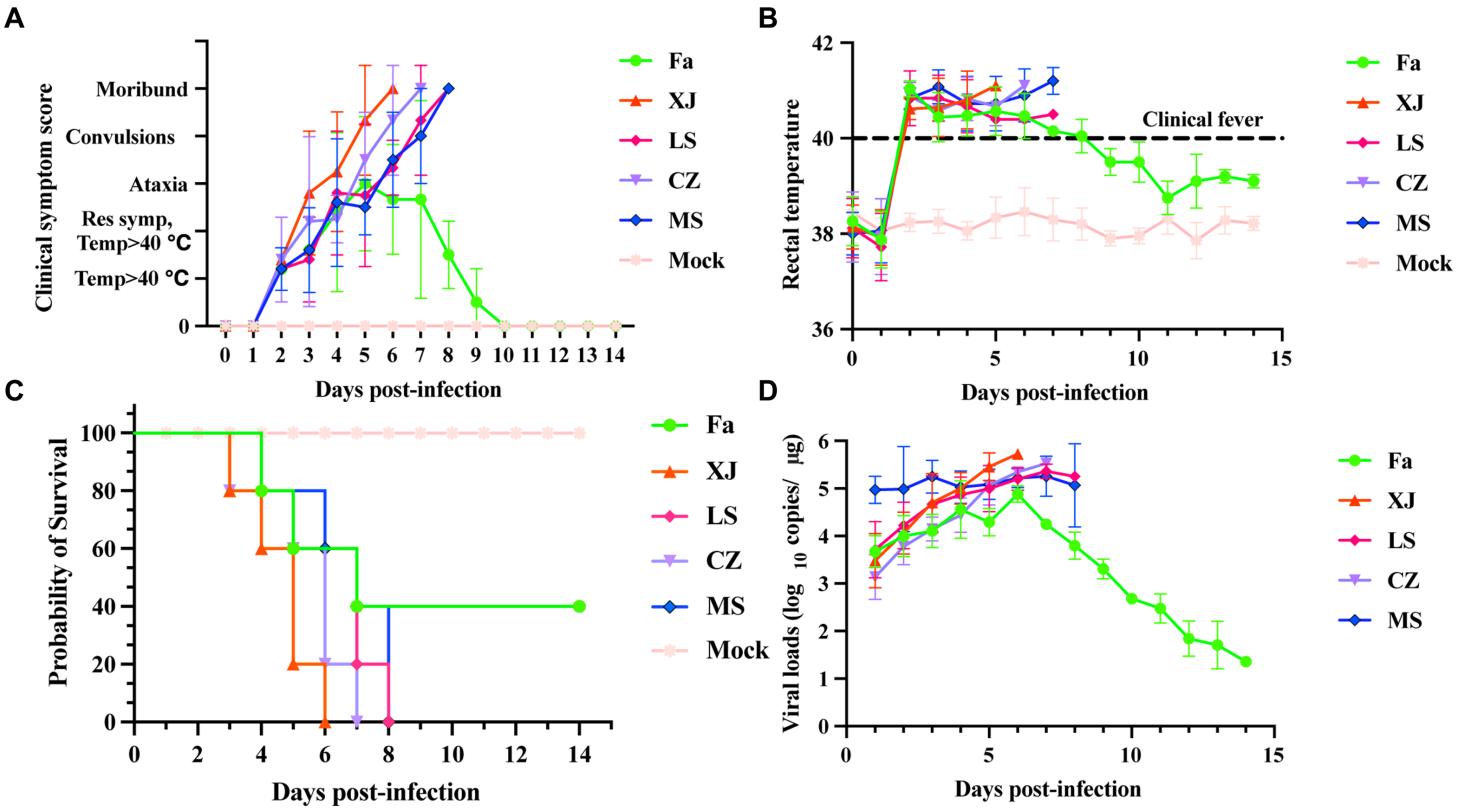
Clinical symptoms and shedding virus of newborn piglets challenged with the PRV strains. Newborn piglets were infected with different PRV strains or infected with DMEM as a mock group. The survival rate, clinical symptoms and shedding virus of each group were observed and recorded daily. **(A)** Clinical symptom scores of piglets after challenge. Clinical symptoms, including body temperature, respiratory distress, ataxia, convulsion, and moribund or dead, were scored as previously described (25). **(B)** The rectal temperature of all piglets after challenge with PRV or DMEM. The newborn piglets were challenged with different PRV strains or DMEM, and then the rectal temperature was recorded daily. Clinical fever was defined as a rectal temperature ≥ 40°C. **(C)** Survival curves of newborn piglets infected with different PRV strains were generated. **(D)** The shedding virus of newborn piglets after challenge. Nasal swabs were collected daily, and the PRV copy number was determined by qRT-PCR. Copies/μg represents the number of DNA copies of the PRV genome per 1 μg of total DNA.

Piglets infected with the variant viruses LS, XJ, CZ, and MS developed severe clinical symptoms such as muscle spasms, depression, decreased appetite, and reduced water intake, resulting in rapid death. In contrast, piglets in the Fa strain-infected group showed mild shivering, depression, and decreased appetite. From the 9th day post-infection, the symptoms of the surviving piglets stabilized and did not progress (Figure 4A). Within 2 days post-infection (dpi), piglets in the variant virus-infected groups developed a fever exceeding 40°C that persisted until their demise. By the 10th day post-infection, surviving piglets in the Fa-infected group had returned to a normal body temperatures range of 38.5°C to 39.5°C (Figure 4B). Piglets infected with the variant viruses LS, XJ, CZ, and MS began to die 72 h post-infection, and all had succumbed within 8 days. In the Fa-infected group, 2 out of the 5 piglets survived (Figure 4C). Daily nasal swab collections were conducted to assess viral shedding, followed by analysis with qRT-PCR. PRV was detected in the nasal secretions of all infected piglets, with peak viral shedding observed on the 6th day post-infection. Compared to other PRV-infected groups, the MS-infected group exhibited higher viral copy numbers from the initial day infection (Supplementary Figure S2). In contrast, the Fa strain showed a declining trend in viral copy numbers following the 6th day post-infection (Figure 4D).

### 3.4 Pathogenicity analysis in piglets

**Figure 5:**
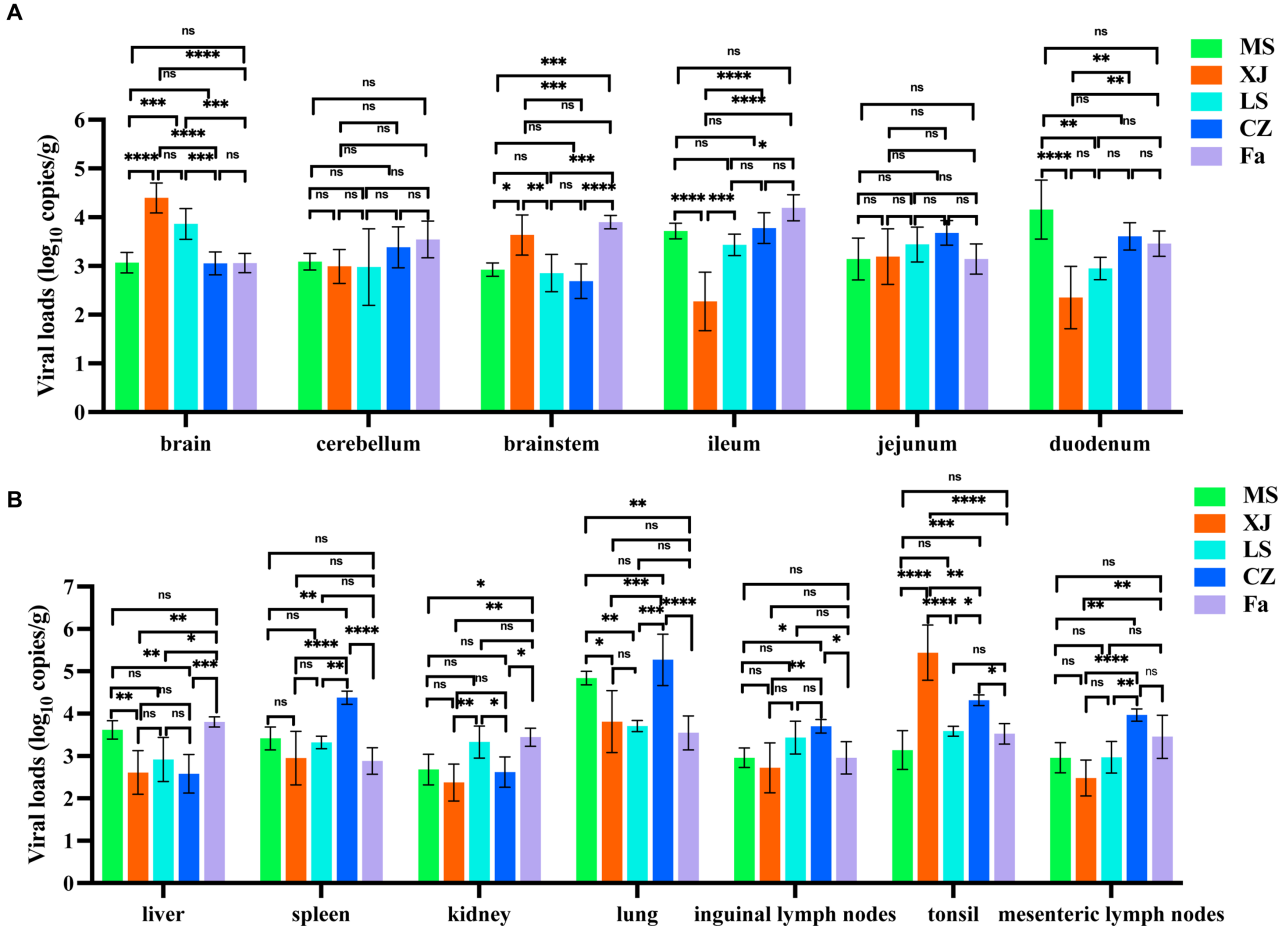
Viral load and histopathological analysis in PRV-challenged piglets. **(A,B)** Viral load of different PRV strains in various tissues of PRV-challenged piglets. Brain, cerebellum, brainstem, ileum, jejunum, duodenum, liver, spleen, kidney, lung, inguinal lymph nodes, mesenteric lymph nodes and tonsil from PRV-challenged piglets were tested to determine the copy number of PRV by qRT-PCR. copies/g represents the number of DNA copies per microgram of tissue. The data in **(A,B)** represent the mean ± SD. Experiments were performed independently with at least three biological replicates. One-way ANOVA was used for analysis. * *p* < 0.5; ** *p* < 0.01; *** *p* < 0.001; **** *p* < 0.0001; ns, not significant.

Quantitative PCR results showed no significant differences in viral loads in the cerebellum and ileum of piglets infected with any of the five different PRV strains. However, strains XJ and LS showed significantly higher viral loads within the brain. Additionally, strains XJ and Fa strains exhibited significantly higher viral loads in the brainstem compared to the other strains (Figure 5A). The CZ strain was observed to have a significant increase in viral loads in lymphoid organs, including the spleen, inguinal lymph nodes, and mesenteric lymph nodes, surpassing those of other strains. In digestive organs, such as the liver, ileum, and duodenum, the MS and Fa strains showed significantly higher viral loads compared to the other strains. In lung tissue, the MS and CZ strains showed significantly higher viral loads compared to the other strains, showing variations in tissue tropism among different PRV strains (Figure 5B).

### 3.5 Cross-neutralization responses between variant strains and classical strains

**Figure 6:**
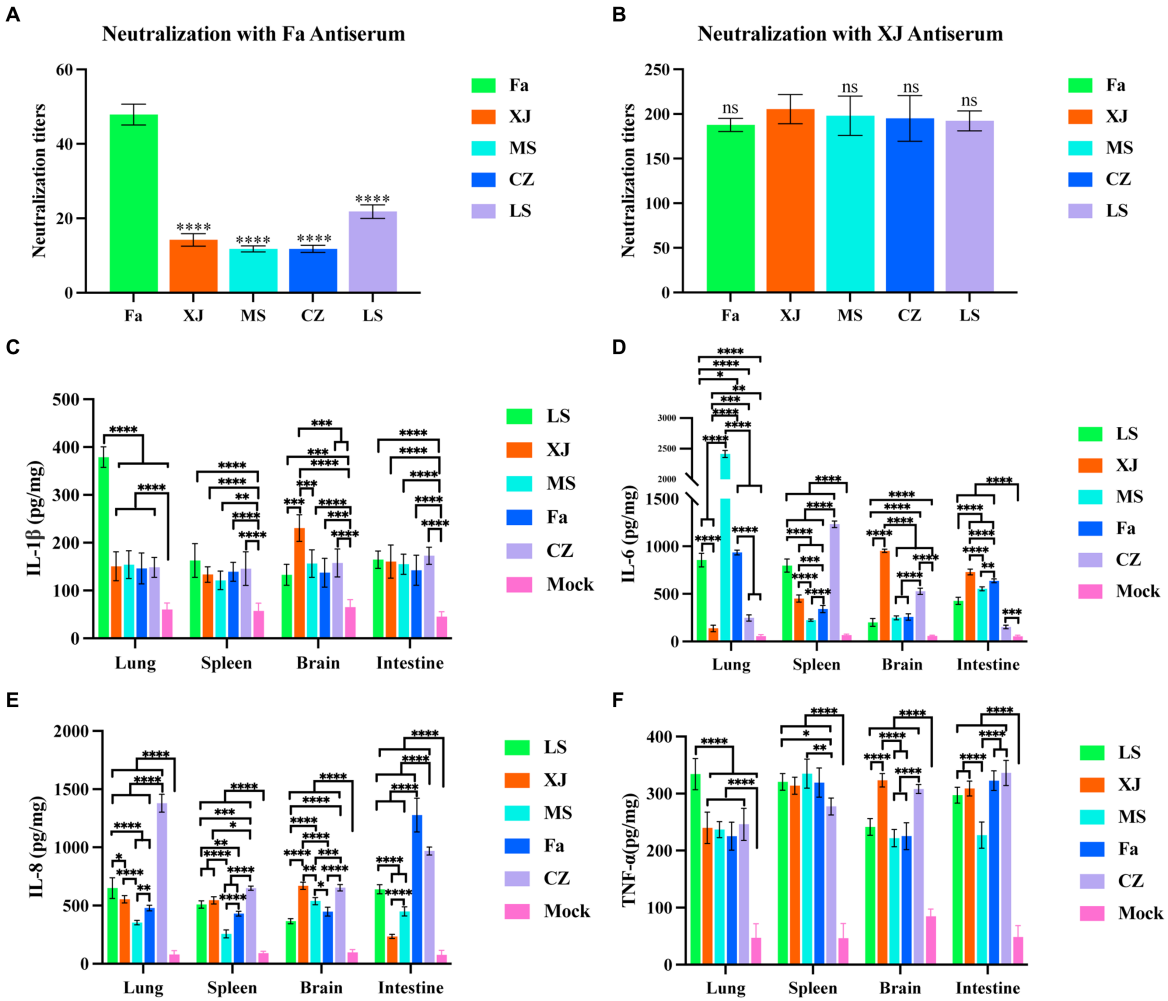
Cross-neutralization tests and inflammatory factor levels in the lungs, liver, brain and intestine. **(A)** Neutralizing titers of Fa strain antisera against the 5 PRV strains. The significance of differences between the Fa group and other groups was analyzed with one-way ANOVA. **** *p* < 0.0001; ns, not significant. **(B)** Neutralizing titers of XJ strain antisera against the 5 PRV strains. The inflammatory factor levels of the tissues in all groups, including IL-1*β* **(C)**, IL-6 **(D)**, IL-8 **(E)** and TNF-*α* **(F)**. The significance of differences between the mock group and other groups was analyzed with one-way ANOVA. ** *p* < 0.01; *** *p* < 0.001; ****, *p* < 0.0001. The data in this figure represent the mean ± SD. Experiments were performed independently with at least three biological replicates.

Subsequently, we performed cross-neutralization assays to evaluate the serological cross-reactivity between different PRV strains. Antisera derived from vaccination with the classical Fa strain showed a significantly reduced neutralizing capacity against the variant strains, including XJ, as compared to its neutralizing capacity against the Fa strain itself (Figure 6A). In contrast, antisera from the XJ strain displayed neutralizing titers comparable to those against the Fa strain for all variant strains assessed (Figure 6B).

### 3.6 Induction of high levels of inflammatory cytokines in tissues after PRV infection

Inflammatory cytokine assays demonstrate that compared to the mock group, IL-1*β*, IL-6, IL-8, and TNF-*α* were significantly upregulated in the lungs, spleen, intestines, and brain post-infection. Specifically, the LS strain induced significantly higher levels of IL-1*β* in the lungs, and the XJ strain induced significantly higher levels of IL-1*β* in the brain compared to other PRV strains (Figure 6C). The MS strain induced significantly higher levels of IL-6 in the lungs, and the CZ strain induced significantly higher levels of IL-6 in the spleen compared to other strains. In both the brain and intestines, elevated IL-6 levels were observed with the XJ strain, surpassing those induced by the other strains (Figure 6D). The CZ strain induced significantly higher levels of IL-8 in the lungs, and both the Fa and CZ strains induced significantly higher levels of IL-8 in the intestines compared to other strains (Figure 6E). Furthermore, the LS strain induced significantly higher levels of TNF-*α* in the lungs compared to other strains (Figure 6F).

## 4 Discussion

PRV, the causative agent of Aujeszky’s disease, has caused significant economic losses in the swine industry since the emergence of variant PRV strains in China in 2011. The three isolated PRV variant strains exhibited indistinguishable *in vitro* proliferation characteristics in BHK-21 cells, with all strains reaching peak viral titers within 36 h, reflecting their comparable replication abilities *in vitro*. But plaque assay results showed that the LS, MS, XJ, and CZ strains exhibited significantly higher plaque generation ability compared to the Fa strain, which may be due to the weaker cell-to-cell spread ability of the Fa strain. Previous studies have found that the deletion of amino acids 124–495 in the HSV-2 gE protein severely inhibits the cell-to-cell spread efficiency of HSV-2 (26). The gE protein, as a membrane protein, is closely related to the cell-to-cell spread ability of PRV, and is also involved in the transport and localization of viral particles, affecting viral evasion and dissemination (27). Distinct from the Fa strain, the MS, CZ, XJ, and LS strains have mutations at aa54 (G to D), aa403 (P to A), aa448 (V to L), aa492 (G to D), and aa518 (S to P) (Supplementary Figure S1). These specific amino acid substitutions could potentially influence the efficiency of cell-to-cell spread in the variant strains. To validate these findings, further studies involving the construction of mutant and revertant strains are required. This will be a focus of our laboratory’s future research efforts.

In assessing the neutralizing abilities of sera, we observed that animals immunized with the XJ strain exhibited similar neutralizing capacities against all variant strains. This suggests that vaccines based on variant strains can effectively prevent infection by prevalent strains. This neutralizing ability may be attributed to the consistency of neutralizing epitopes on the gB protein between variant strains and the classical strain (28). In contrast, neutralizing antibodies derived from the classical strain showed limited effectiveness against variant strains, possibly because the variant strains have evolved immune escape mechanisms, including the evasion of neutralizing antibodies induced by the classical strain. Ren et al. (29) found that gC and gD proteins contribute to evading neutralizing antibodies induced by the Bartha-K61 vaccine. Deng et al. (30) discovered differences in the usage of immunoglobulin heavy chain variable region genes and the combination of immunoglobulin heavy chain variable region and IGHJ genes between the Bartha-K61 and XJ strain infection groups. Additionally, PRV has developed mechanisms to evade host innate immunity, such as UL13 suppressing the expression of RIG-I and MDA5 by inhibiting the activation of the transcription factor NF-κB (31), and gE counteracting cGAS/STING-mediated IFN production (32). These mechanisms may differ between classical and variant strains. In conclusion, we hypothesize that PRV variant strains may have evolved multiple mechanisms to escape neutralization by antibodies against classical strains, but further research is needed to confirm this.

The different strains of PRV induced varying levels of cytokines in specific tissues, reflecting their unique pathogenic profiles. For instance, the LS strain’s induction of high IL-1*β* and TNF-*α* levels in the lungs suggests it may cause more severe pulmonary pathology compared to other strains. In addition, IL-1*β* is crucial for the activation of inflammatory responses, and TNF-*α*, a key mediator of inflammation, should exhibit trends consistent with IL-1*β*. This is consistent with our findings. The XJ strain’s significant induction of IL-1*β* and IL-6 in the brain implies a propensity for causing severe neurological damage, aligning with observed clinical symptoms like muscle spasms and depression. Several studies have indicated that encephalitis is a major cause of mortality in infected animals (33–36). However, in the surviving piglets of the Fa group, elevated levels of inflammatory cytokines were also detected in the lungs, brain, intestines, and spleen compared to the control group. Therefore, the mechanisms underlying acute mortality in piglets caused by PRV remain to be further elucidated.

Our study has several limitations. First, the limited number of PRV strains, all from specific geographical locations, may not fully represent the genetic diversity of PRV. For instance, we cannot determine whether there are significant differences in the onset, course, and pathogenicity of diseases caused by early variant strains compared to currently prevalent variant strains. Second, our *in vivo* model was restricted to piglets, which may not comprehensively capture the disease dynamics in other pig populations. Future research should address these limitations by including a more diverse set of PRV strains from different regions. Additionally, exploring other animal models and conducting longitudinal studies will provide a more comprehensive understanding of PRV pathogenesis.

## Data availability statement

The original contributions presented in the study are included in the article/Supplementary material, further inquiries can be directed to the corresponding authors.

## Ethics statement

The animal studies were approved by the Ethics Committee of Sichuan Agricultural University. The studies were conducted in accordance with the local legislation and institutional requirements. Written informed consent was obtained from the owners for the participation of their animals in this study.

## Author contributions

LX: Conceptualization, Data curation, Formal analysis, Software, Writing – original draft, Writing – review & editing. QT: Formal analysis, Software, Writing – review & editing. TX: Formal analysis, Software, Writing – review & editing. YY: Supervision, Writing – review & editing. YaZ: Supervision, Writing – review & editing. ZL: Formal analysis, Writing – review & editing. YuZ: Software, Writing – review & editing. LZ: Conceptualization, Funding acquisition, Project administration, Writing – review & editing. ZX: Conceptualization, Resources, Validation, Writing – review & editing.

## Footnotes

^1^ https://ictv.global/taxonomy
