# Supplementary material for: Pathogenicity characteristics of different subgenotype pseudorabies virus in newborn piglets
Source: Front Vet Sci. 2024 Aug 7;11:1438354. doi: 10.3389/fvets.2024.1438354 (PMC11335603; doi:10.3389/fvets.2024.1438354)
Supplement: Supplementary file 1 [file Image_1.pdf]

Supplementary Material

1 Supplementary Figures

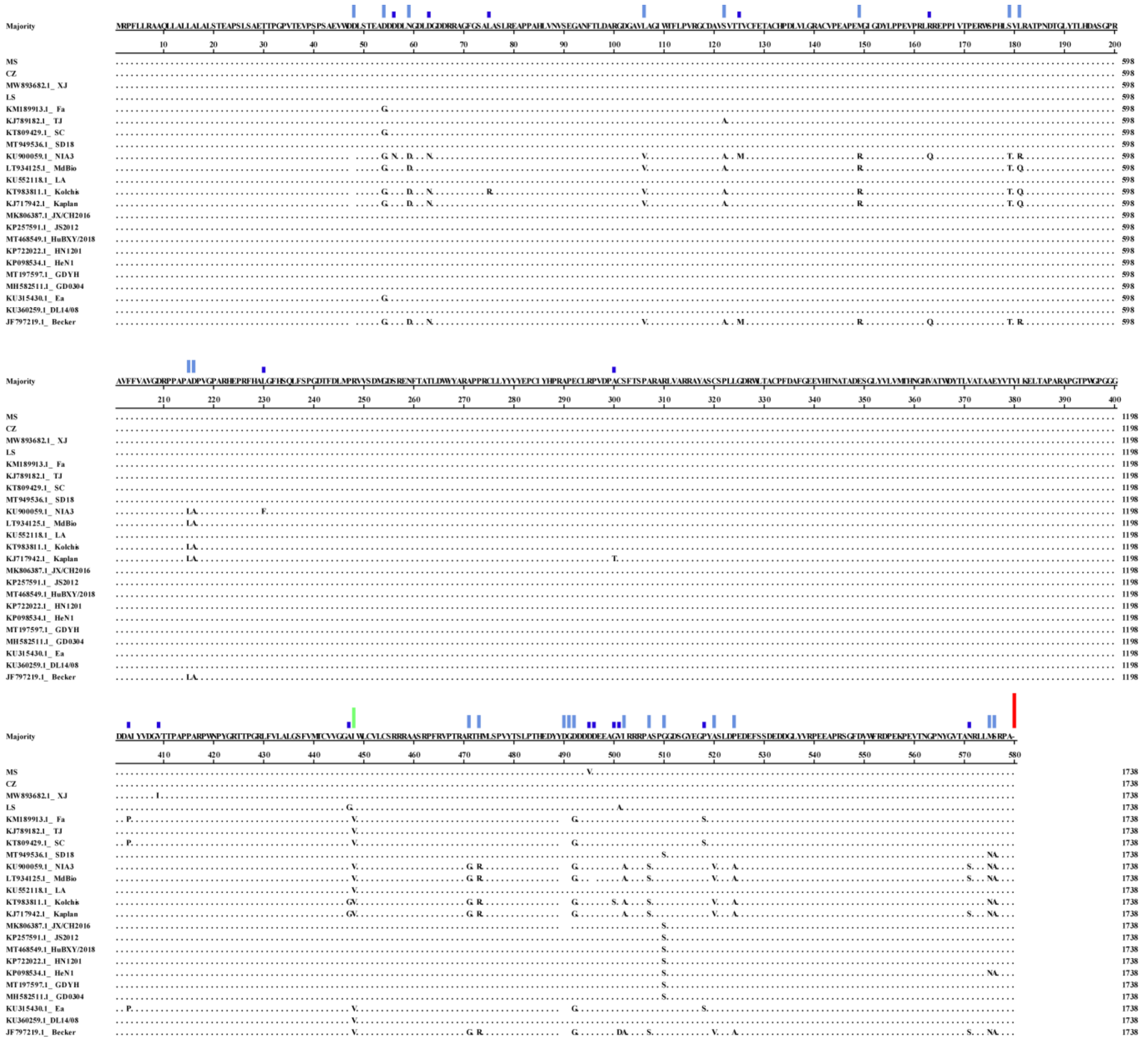

Supplementary Figure 1. Amino acid analysis of gE protein.

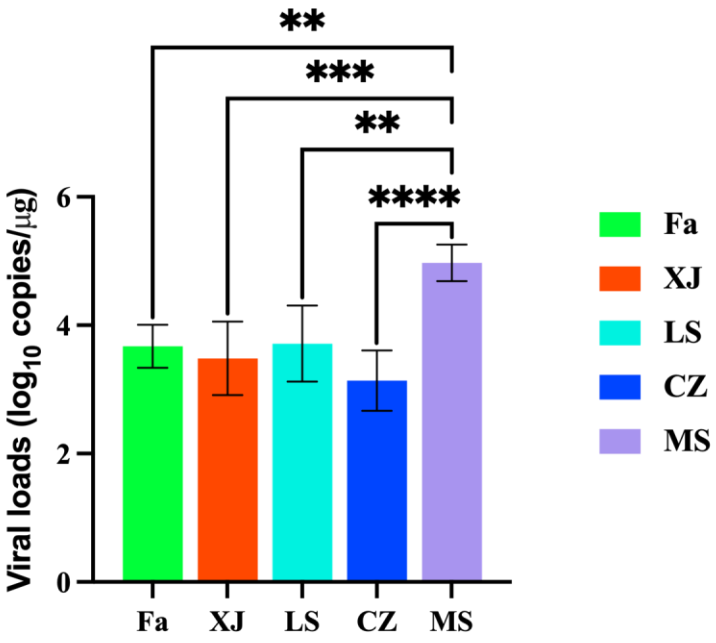

Supplementary Figure 2. Viral Shedding on Day 1 Post-Infection.
